# Syphilitic Lesions of the Viscera

**Published:** 1872-07

**Authors:** James N. Hyde


A MONTHLY RECORD OF

*Medicine, Surgery and the Collateral Sciences.*

EDITED BY J. ADAMS ALLEN, M.D., LL.D.; AND WALTER HAY, M.D.

VOL. XXIX. — JULY, 1872. — NO. 7.

### Original Communications.

ARTICLE I. — *Syphilitic Lesions of the Viscera.* A Paper read before the Chicago Society of Physicians and Surgeons, by JAMES N. HYDE, M.D.

It is a pathological fact, as curious as it is interesting, that the field of certain forms of inflammation coincides with the field of certain ultimate structural elements; and that the boundary lines of this field are, oftentimes, traceable only by minute differences of cell-formation. How often, for example, is the mucous wall of the fauces affected by an inflammation which never encroaches upon the membrane of the larynx; or the cavity of the prepuce filled with the infectious discharge of a balanitis, while the urethra remains intact. In the former case, the edge of the epiglottis, in the latter the lip of the urinary meatus, has an elementary structure, different, though slightly different, from that of the surrounding parts; and this difference offers an effectual barrier to the spread of the disease. There are, nevertheless, inflammations of such violence and virulence that they overleap the limits respected by the milder grades, and encroach upon every portion of the human economy. The question as to whether a given inflammation will exceed a given boundary, is one which involves the degree, rather than what is called by the French, the "*specificity*," of the disturbance; and this is a consideration of importance in investigating the "*Syphilitic Lesions of the Viscera.*"

If there is any disease in our nomenclature, whose natural history can be accurately described, and whose bearings, if I may so speak, we can, at any period of its career, ascertain by the chart, such a disease is syphilis. Unlike almost all others, the date of its inception is exactly determinable; no doubtful prodromata confuse its immediate diagnosis; every one of its stages furnishes its own special signs of existence; and its issue, when left to itself, is almost capable of prediction. In the course of this its natural and unmodified career—the field which it selects for the display of its phenomena, possesses a striking importance from the very uniformity of its predilection. As a rule, those tissues and organs are invaded which are developed from the external serous or animal layer of the blastodermic vesicle. The entrance of the virus is effected at the superfices of the body. Then, in succession, it attacks the external integument and the apparatus accessory to it: the constituent elements of the skin, the nails and hair, the sweat and lymphatic glands, the muco-cutaneous tissues, the eyes, nares, mouth, and external genital. Then the locomotory apparatus suffers—in cartilage, bone, tendon, muscle, and periosteum. Finally, as if by one saltus, the disease passes over to its *inheritor*—here again affecting the external integument, often reappearing at the anus—and repeating, in its hereditary form, many of its old excursions into tissues already indelibly marked by scars in the progenitor. Is it not almost the exception to discover in the history of this career, any visceral complication—any passing over, as it were, of that structural limit already described—any transference of its destructive agency, from the organs developed from the external animal layer, to those originating in the internal mucous or vegetative layer of the human blastoderm?

Witness, for example, the patients in any venereal ward of a hospital. There you will find some exhibiting every stage of the disease—some with carious ulceration of the cranium—others dying from destructive disorganization of the soft parts of the face, throat or limbs; and not one case of *visceral syphilis.*

This is the more remarkable in view of the readiness of such affections, attended with cutaneous eruption, as the variolous, the scarlatinal, the erysipelatous and the rubeolar, to assume aggravation in consequence of inflammation of the lungs, brain or kidneys. And the literature of syphilis attests the same fact. Here visceral lesions are more or less accurately described, but illustrative cases are rarely given, which might enable the reader to gain exact ideas of the complications, or the descriptions are so vague and general that the student comes to regard them as possibilities to be specially examined, when, if ever, they occur in practice.

Subjoined are the outlines, for which I have to depend largely upon my memory, of the only two cases of visceral syphilis, that have occurred to my personal observation during ten years' study of the disease—some of them passed when in charge of venereal wards. The first of these cases was treated in hospital, and the second presented itself in private practice but a few weeks since.

The hospital patient was an adult male, thirty-two years of age, brought into the medical ward with a card from a surgeon, stating that he was affected with "pulmonary tuberculosis." It was added that he was losing flesh, that moist rales were audible in his chest, and that the most distressing symptom in his case was his excessive *hæmoptysis.* Of this latter fact there could be no doubt, as I had an opportunity of verifying it—not once but often, while he was in my charge. On the very afternoon of his admission, I witnessed at his bedside a discharge of blood greater than I had ever seen escape from a consumptive. He was half-erect in bed, his frame was extraordinarily convulsed in the paroxysms of the cough, his face suffused with color, and he held before him a hospital basin almost full of bright-red blood, mixed with the saliva and mucus expelled by the same violent effort. I administered Monsel's salt by insufflation, i. e., locally and by the mouth, till the hemorrhage ceased, and then made a careful examination. He was large, and had ample chest-room. The bony framework of his thorax was unusually well developed, and his complexion was clear, without either that transparent delicacy peculiar to one class of phthisical cases, nor the dirty, earth-discoloration characteristic of another. He was thin but not emaciated—his thinness being rather that apparent in large-boned men, where the osseous projections contrast with the surface hollows, and which is not due to disappearance of the fat-cells. Percussion of the chest elicited a distinctly normal sound on both sides, except over a small space on the right, between the third and fourth ribs, extending between a perpendicular line passing through the nipple and another dropped from the anterior border of the axilla. Auscultation there detected an absence of the respiratory murmur, and in the neighborhood were occasional distinct rales, due to the effused blood and mucus in the larger tubes—partly, no doubt, resulting from the violent effort made in coughing. On the left side, the respiration was normal. The pulse numbered 80 per minute, and the respirations 20; the pulse-respiration ratio being 1–4, and slightly raised, as I subsequently discovered, above the proportion which was in his case normal—the difference being due to the excitement consequent upon hemorrhage. His tongue was clean; there was no perversion of appetite, bowel function, or sleep, and consequently no night-sweating. I left him quiet, with directions to renew the hæmostatic treatment, if the necessity for it recurred; and I might add, that I left him completely ignorant of the nature of his malady. After carefully excluding the possibility of the existence of pulmonary phthisis, thoracic aneurism and apoplexy of the lungs, I was at a loss to account for his symptoms.

The clue to the case came to me by accident. I was stating its singular features to Dr. C. D. Maxwell, when he asked to see the patient, and, upon being shown him, recognized, at once, a former inmate of the venereal ward, discharged but a few months previously, after treatment for secondary syphilis. The patient had, up to this time, denied the fact of previous disease, but now admitted the deception. Dr. Maxwell stated to me, that he, the patient, had been relieved of a syphilitic eruption, so general that it extended to the tips of his ears and toes, and so vivid that it gave him the appearance of a "boiled lobster." I suggested that his present disorder might have originated in his late attack, but Dr. M. thought otherwise, and expressed to me his belief that the man was really consumptive and would die in a few months.

I was convinced, however, that there were gummy tumors of the lung, induced by syphilitic infection, which were softening, ulcerating, occasionally opening into a blood-vessel, and located at that point of the chest where the morbid phenomena were evident. The line of percussion-dulness distinctly defined this space. Around it, during the interim of the hemorrhages, the ronchi which were audible when the air-cells and tubes were partly filled with blood and mucus, gave place to an entirely normal respiration. Over it, there was no indication of a respiratory murmur.

I determined on specific treatment soon after this; not immediately, as I was fearful of exhibiting remedies which might impair the plastic quality of the blood, and hasten the disintegrating process. But soon this became necessary, as nothing else relieved him. Every other day—or about as often—the same violent hæmoptysis would recur; in each, there was an astonishing quantity of blood ejected, and in each, the same violent cough convulsed the chest and entire frame, as if in the effort to expel an irritating foreign body. When relieved by the hemorrhage the cough disappeared entirely; and there resulted a pulse, perhaps somewhat softer than before, moderate weakness, a fair appetite, and the primæ viæ in a normal condition.

I commenced by administering five grains of the iodide of potassium tentatively, and slowly increased this dose to a scruple, three times daily, given without combination and in abundant solution. Iodism was soon induced, with coryza, and the characteristic eruption appeared, mingled with some copper-colored blotches around the nuclea. The medicine was suspended, then resumed in smaller doses, and an interval of ten days occurred, without pulmonary hemorrhage—the longest since his admission—the patient meantime gaining in flesh and strength, and manifestly improving in appearance. Then occurred another severe hæmoptysis, which was followed by the large doses of the iodide; the flowing of blood having been arrested in this, as in his later attacks by the use of an infusion of matico, which I had seen recommended for that purpose, and which relieved him more speedily than the method first used. Then ensued another long interval of repose, without hemorrhage, during which the space which was dull under percussion, slowly contracted its limits, and became less easy of recognition. A thin stratum of respiratory murmur, if it may be so described, interposed itself between the ear and the subjacent condensation of the lung-tissue. This gradually extended and deepened. In fifteen days more, the last hemorrhage occurred, and it was insignificant in character and amount. I concluded by adopting a mercurial course, internally; and externally by the improvised vapor-bath, given with the lamp, chair and blanket. As the case was interesting, I retained the patient in hospital longer than was customary, to make sure of the issue. The cure seemed positive and final. He was afterward discharged, with every appearance of robust physical health, and six months later when I saw him, he assured me of his continued improvement.

With regard to the remedy which seemed to prove of the greatest value to this patient, it is necessary to make an explanation. Dr. F. J. Bumstead—who is deservedly esteemed the most eminent of American syphilographers—taught, when I first listened to his lectures, that the iodide of potassium was ineffectual in syphilis, except when administered in the tertiary stage, and that then its chief use was in eliminating mercurial preparations which were presumed to be residual, or left fixed in the system, under one form or another. It was noticed that ptyalism sometimes occurred when the iodide was exhibited after a mercurial course, and it was inferred that the former remedy combined with the latter in some way, so as to free it, and render the new compound capable of elimination by the secretory and glandular system. Hence the iodide was to be used in tertiary syphilis only, and then after a mercurial medication.

My own convictions have compelled me to reverse, or rather accept inversely, the advice of that day. Experience has taught me that when syphilitic patients in any stage of the disease present themselves for relief—and they often present themselves when they require *speedy* relief—the iodide of potassium is the one remedy which will most surely and most speedily procure that relief; and that the mercurial is often indispensable in order to make that relief permanent.

At that time, also, the ordinary dose of the iodide for an adult was ten grains. That was the conventional, the routine, dose. I had seen the remedy used in numerous hospital patients, sometimes in those inveterate cases which are the despair of the outgoing house surgeons, and the subjects of experiment for all the incoming newly-graduated staff, but I had never known that dose to be exceeded. It remained for me to obtain from a friend who studied his profession in Paris—an intimate of the French philosopher, Cousin—and something of a philosopher himself—a knowledge of the power possessed by this drug. I believe, to-day, that it will control the extension of the syphilitic virus *for a time*, as completely as quinia will check the miasmatic influence.

The circumstances were these: A patient in Bordeaux, France, with whose symptoms I was familiar, had been carried to his room in the hotel on a litter, in consequence of periosteal disease in both tibiæ, for which he had been undergoing a tedious mercurial treatment. In the course of a few days, during which he received, for the first time, the care of my friend, the doctor, he improved so much in general health and spirits, that he attended a ball where he joined in a waltz. I inquired of his physician, how he had effected such a charm. His reply was short, "*Enormous doses of the iodide!*" He went on to say, that he had "learned when living in the Quartier Latin, to use the remedy in this way, commencing with the usual dose and increasing it cautiously to a scruple, drachm and even half an ounce, in well-diluted solution." I had heard of this before, but the practical effect just wrought with the aid of such doses, gave me a vivid idea of their possibilities of usefulness.

This was in 1866. In October of 1871, Dr. Bumstead, in an interesting article, which appeared in the *American Journal of Medical Sciences* of that date, page 578, and which was reprinted from the September No. of the *American Practitioner* of the same year, offered some new suggestions on the treatment of syphilis. I quote his words: "Relief will be had, and important organs will be saved, by giving one hundred grains (of the iodide of potassium) when the disease only laughs (metaphorically speaking) at fifteen or twenty! Patients find this out themselves, when you have not stinted them in the use of the remedy, and will tell you, as one of my patients with syphilitic necrosis of the ulna recently did me, that forty grains three times a day had no effect, while fifty three times a day were at once followed by a manifest improvement. The iodide of potassium has been given with impunity in the quantity of two or three ounces in the 24 hours for several weeks, and even months, but this amount is unnecessarily large. I have never had occasion to exceed three drachms per day, and from a drachm and a half to two drachms is usually sufficient."

Dr. B., however, still confines its use to *tertiary syphilis*, and then employs coincidentally a mercurial inunction. I have not seen the edition of his work just completed, and do not know if this improvement in treatment is there noted.*

In connection with this subject, I condense from the Frevel of Lancereaux, the details of a case which I believe does not appear in the abridged translation of his voluminous treatise:

Louise R——, a laundress, 41 years of age, states that her father and mother, and nine of their twelve children, have been syphilitic. At about eight years of age, she became partially blind, and had sore throat and aphonia. At fourteen, occurred a deafness which disappeared, then returned, and still persists. At 18, she had not menstruated, and suffered from slow fever, intense headache, vertigo, and alopecia. In 1859 she had pleurisy; and two months later, entered hospital, where she suffered from hæmoptysis, repeated at the close of that year, and the beginning of the next. In June of 1860, when in fair health, she had an exceedingly abundant hæmoptysis, and estimates the quantity of blood passed in 24 hours, at one litre.

She is a small, scarcely-developed woman. Her breasts are those of a young girl not arrived at puberty; her vagina scarcely permits the introduction of the little finger. There is no trace of a hymen or its relic; her voice is harsh; her nose flattened at the base; her head almost bald; her face pale, but not emaciated. A recent hæmoptysis, with vomiting of food and cod liver oil (which she takes, habitually, in large quantities) are the reasons assigned for her applying to hospital. She has pain in the shoulder and arm of the right side, and some distress in the stomach. Deafness exists to such an extent that questions have to be addressed to her in writing. There is no alteration of the external ears. Percussion dullness and obscure respiration are discovered over a space several centimetres in extent, above and internal to, the right breast; at the same level, and further toward the axilla, a soft and impulsive souffle is audible, quite different from the bronchial sound, and possessing *inferiorly* a hollow quality. Occasionally, during cough

* All the editions of Dr. B.'s work mention these large doses as in use, but their practical value is not insisted upon!

a profound inspiration, subcrepitant or cavernous rales are audible. Posteriorly, these phenomena exist, but are less distinct. There is frequent cough, with abundant bloody expectoration; the sense of smell is almost entirely destroyed, and her appetite is deranged, with marked gastric disturbance, and hectic with evening exacerbation. Flying blisters were applied to the chest, and a soothing potion administered. This condition continued—with moderate appetite and progressive emaciation; her face remaining pale and full—during November and December; when she had numerous returns of hæmoptysis. She left the hospital in January.

In March she returned still more emaciated, with persistent cough and habitually sanguinolent expectoration. The chest signs are: cavernous souffle a few lines from the clavicle, audible but less distinct posteriorly; and percussion dullness over the same region. Occasionally loud gurgling mucous rales were heard. The fever continued, increasing in intensity, with anorexia and diarrhœa. She died in 1861 after progressive marasmus.

At the autopsy *no trace of tubercle* or *tuberculous* disease was *discovered* in the chest. The right lung contained several cavities produced by ulceration in the midst of a tissue extremely indurated. Some of these vomicæ would contain a pigeon's egg, and had smooth, shining walls. These indurated portions had a greyish appearance, and were so tough they could not be torn.

At the autopsy of a similar case, reported by this author, where hæmoptysis had also been a prominent symptom, these indurations were said to resemble marble of a sea-green hue, and to have the feeling of a periosteal node. No tubercle was anywhere discovered.

In considering the symptomatology of these cases, we ask, to what extent are they induced by syphilis? and, how far are they merely the result of a cachexia, brought about by syphilis it is true, but in their issue like those produced by other depressing agencies? There can be but one reply. The treatment, or rather the result of the treatment, is a key to the problem. Could a patient suffering from tuberculous consumption, find relief and restoration to health, in a few weeks' course of the iodide of potassium and mercury? Would any one expect to find in a woman dead of tuberculosis of the lung, after years of suffering, pulmonary cavities as large as pigeon's eggs, without their usual purulent, disintegrated and ragged parietes?

Walshe discusses this deposit in one short paragraph. "The syphilitic gummata of the lung," he says, "look like nodules of lobular pneumonia—they soften and are eliminated—are non-vascular, and have a consistence rivaling that of scirrhus. Their diagnosis must be difficult. The physical signs are those of solidification, softening and excavation—local and general symptoms simulate phthisis. There is strong reason to believe the iodide of potassium essential. Precise information is wanting."

My attention has been recalled quite recently to the visceral complications of syphilis by the only other case falling under my observation, which occurred in private practice a few weeks since:

Mr. S——, a member of the Chicago Board of Trade, married, without children, and in very comfortable circumstances, applied to me one year ago, with synovial inflammation of the ankle-joint; relieved, after assuming a particularly sluggish phase, by rest, leeches, and the application of a leathern splint. While under treatment I discovered two pustules of ecthyma on the scalp, and he exhibited to me a small indolent ulcer, as large as the head of a carpet-tack, over the first tarso-metatarsal articulation of the right foot. He then admitted, on questioning, the existence of a single chancre two years previously, and before his marriage, when he was in St. Louis, which had been cauterized by a physician of that city, who had also given him some medicine, and assured him of no future ill-consequences. Since then he had been treated by the late Dr. C. C. P. Hildreth of our city for over a year, for chronic catarrh and slight deafness, due to inflammatory obstructions of the Eustachian tube. I advised him to undergo specific treatment, but the demands of his business were pressing, and the returning usefulness of his ankle-joint enabled him to return to his duties. Meantime his wife was delivered of a blighted ovum.

He returned to me with a syphilitic onychia, located at a point where he had received previous injury from a bruise, viz., over the second toe of the right foot. Near this, was the small ulcer which has been already referred to, now enlarging and deepening. On the occasion of the previous treatment, he had given me opportunity to cauterize this but once. Pus had accumulated under the corrugated and deformed nail, and its matrix was sloughy and ill-looking. I completely removed the nail, with the scissors, and cauterized its bed most thoroughly, as well as the neighboring ulcer, dressing both with a reduced ointment of the nitrate of mercury. An abscess formed in the instep of the same foot, which discharged, when opened, a very ill-conditioned pus. This was treated with caustic and similar dressings. Mr. S. then took internally the iodide of potassium, in doses which were gradually increased to about one drachm daily. He also rubbed into the axilla at night, a quantity of mercurial ointment as large as a bean. This was continued for a week, when the quantity of the iodide taken amounted to more than a solid ounce. No ill effects had been produced, slight iodism had occurred, but the ulceration, which before had steadily extended, now gave place to simple granulating wounds. A course of the protiodide of mercury was then substituted for all other medication, and would have been steadily persisted in, had not the wounds of the foot completely healed, and the patient, as on the previous occasion, considering himself quite cured, returned to immerse himself in a business which left him no time for medical consultations.

After the great fire, his engagements for a time gave him opportunity to consult me with reference to his minor ailments. He soon appeared complaining of moderate anorexia, and I gave him a little ferruginous tonic. His appetite began to improve soon after, but he returned and stated that he suffered from some sweating at night, and that his general health was poor. Yet his skin was clear, and there was no trace of any recurrence of his former trouble. I confess that while I admitted to myself the possibility of a syphilitic dyscrasia, I hesitated to resume a debilitating treatment, with no positive indication of its necessity. Especially did I dread such treatment when apparently counter-indicated by the night-sweating. I therefore gave him some sugar-coated granules of the dried sulphate of iron and requested him to return in a week. At the expiration of that time he reported himself improved, but his face was quite pallid and there was a manifest puffiness of the left upper eyelid. I examined his urine at once, and that examination threw a flood of light upon his case. Albumen was precipitated by heat and nitric acid, in quantity about equal to one-fifth of the bulk of urine in the testtube. Under the microscope, granular, epithelial casts came into view—some quite large, all perfectly distinct, and markedly granular. The field was tolerably well covered with pus cells. There were no other abnormal ingredients. It was evident that a syphilitic inflammation had involved the pelvis, tubes, and perhaps also the adjacent parenchyma of the kidney. In selecting a mode of treatment it was clear that the same doses of iodide, which the patient had tolerated so well before, might fulfill *now* another indication, in consequence of the elimination by the kidneys; I therefore prescribed the iodide of potassium in scruple doses—the patient taking nearly one drachm and a half per diem (note!) in abundant solution, and, at a different hour, three times daily, twenty minims of the muriated tincture of iron. I looked upon the case, however, as a grave one, and represented it so to him, urging upon him the advantage of a consultation for his own sake, as well as for the confirmation of my views. He named Dr. N. S. Davis of our city, and together we examined the patient. Dr. Davis agreed with me fully as to the diagnosis and treatment, but offered a suggestion as to the medication to be pursued, which I give in full, as it may prove of value to some who are present.

Dr. Davis stated to me that ten years ago, the preparations of pareira brava in market were of such quality that he and his professional associates of that date made efficient use of them in cases of purulent or semi-purulent discharges from the kidney. That of late years the sensible effect of the remedy had diminished, or disappeared, to such an extent that he and others had discontinued its use. The matter had been investigated by the well known New York druggist, Dr. E. R. Squibb, who discovered that only twigs and small branches of the cissamhelas pareira were imported from South America, and he at once proceeded to import a quantity of the *root.* From this he prepared a fluid extract of such quality that he felt confident it would answer all indications formerly fulfilled by the remedy. This preparation Dr. Davis suggested that I should add to the solution of potass, which I was then administering.

I should add, that I have examined a specimen of this fluid extract, for which I am indebted to Mr. Sargent, the druggist, and that it possesses a decided difference in color, taste and other peculiarities from the ordinary extracts.

My patient was then placed upon the following active course of medication: twenty minims of the muriated tincture of iron before each meal; a scruple of the iodide of potassium, and a fluid drachm of the pareira extract after each meal. This course was pursued for one week, during which I examined the urine carefully, and had the satisfaction to observe a daily diminution of the quantity of albumen, till there was left but a faint trace. After taking about two ounces of the iodide, slight iodism occurred, and the characteristic eruption spread over the neck and face. The puffiness of the eyelid, the waxy hue of the skin, and the albumen in the urine, were not distinguishable at the end of two weeks. The treatment had, in the meantime, been changed to smaller doses of the tincture of iron, and one-fourth of a grain of the protiodide of mercury taken with meals.

My patient, who had been greatly depressed on learning that he had a disease of the kidneys induced by syphilis, now became rapidly convalescent. His night sweats had ceased, his cheeks exhibited some color, and at last he assured me he had not felt so well for two years. The case resulted in complete restoration to health. I have seen him several times since dismissing him, and he reiterates the statement that he has not felt so well since his infection.

The last case to which I refer, I translate from Roger, and condense as much as possible. It seems to illustrate the fact that the value of the iodide in syphilis does not depend, in any measure, upon the elimination of mercurial compounds previously administered; as here no mercurial treatment had been previously pursued. Experience, after all, is the great teacher—and here is an "experimentum crucis:"

P——, a woman of fifty-two years of age, was infected by a syphilitic husband, and had suppurating axillary bubo, which left an indelible cicatrix; alopecia; tibial and femoral ulceration; emaciation; pale, earthy discoloration of the skin; epigastric and cephalic pain—the latter often of a grinding character—with nocturnal exacerbation and insomnia. She was admitted to hospital in May, 1863; irregularities and nodosities of the right lobe of the liver, with some tenderness, were then established. The urine was pale, and contained a notable quantity of albumen. It was voided in normal quantity. Under the microscope, granular cells and granular globules (these were almost, of certainty, pus cells) in large numbers, came into view. They were recognized as proceeding from the uriniferous tubules of the kidney.

On the third of June, Dr. Potain administered 2 gramme-(30-grain) doses of the iodide of potass. The cephalic and hypogastric pain disappeared; the tint of the skin improved; the ulcers commenced to heal; sleep, muscular force and embonpoint returned. The liver diminished in volume, but retained a slight tenderness on pressure; and the urine continued to give an abundant albuminous precipitate.

On the 18th of June, the albumen had entirely disappeared, but the abnormal microscopical elements were visible in the urine.

The case terminated in a perfect cure; all traces of renal, hepatic and syphilitic disorder having been succeeded by the usual conditions of sound health.

This is the only case of syphilitic albuminuria, beside the one first given, which I can find recorded. In Jocceni's monograph on Albuminuria, the possibility of a syphilitic cause is not entertained; and in Aitken's article on the same subject, there is merely brief allusion to the two pathological products—interstitial hyperplasia, with fatty and lardaceous degeneration—and small, round, white gummata, with softened centres. But it is added, that "Dr. Dickinson can find no proof of the existence of syphilis as a cause of Bright's Disease." On comparing with this, the chapter on Syphilis in the same work—"Aitken's Science and Practice of Medicine"—the posology recommended contrasts with that to which I have given pre-eminence. The treatment by the iodide is barely alluded to, and the largest dose recommended is fifteen grains in twenty-four hours, (say five grains three times daily,) "gradually increased." Increased to what extent? Would an ordinarily cautious practitioner, relying upon this authority, venture to administer more than 10-grain doses?

Here again we are confronted with the question, To what extent are these visceral disorders due to the ordinary causes of pulmonary, renal or hepatic disintegration, and to what extent are they specific lesions of a specific disease? Here, as before, the treatment resolves the problem. Doubtless, it is possible that the syphilitic cachexia may destroy life by inducing low forms of inflammation of the viscera, just as the intercurrent pneumonia of typhoid fever, or the diarrhœa of consumption, where there is no intestinal tuberculosis, may do the same. But these inflammatory troubles are not connected with gummata or interstitial hyperplasias; in them, specific treatment might hasten the inevitably fatal issue. But the pathological deposits of syphilis in the viscera, are readily relieved by appropriate treatment, if the foregoing cases teach aright.

The rarity of these phenomena with us may depend somewhat upon the circumstance that in our country virulent syphilis is not very common. While there may be, numerically, as many cases of the disease with us as elsewhere, yet all classes of society resort to treatment early in the United States, and the disease is oftener arrested before all the organs of the body have been affected.

A single fact may illustrate this contrast in the prevalence of the disease. Primary chancres in Europe may be discovered in an incredibly large proportion, upon the hands, lips, ears and toes. Prof. H. H. Smith, of Philadelphia, informed me that he had seen but one facial chancre in his entire practice in this country. I have only seen chancres of the hands in ironed men on shipboard; their manacles confining their hands immediately over the genitals, and as cleanliness was impossible, infection of the former speedily ensued.

In conclusion, the etiology, pathology, diagnosis, morbid anatomy and treatment of these affections have been in turn briefly alluded to, as far as the desultory character of these remarks would permit. The length of this paper, extended already beyond its intended limits, precludes the discussion of other syphilitic visceral lesions, and especially that of the liver, whose morbid phenomena in this regard possess peculiar interest.

The consideration which I have endeavored to bring permanently forward, is the great value of the iodide of potassium, administered in such doses as will fully influence the system. For this purpose, small doses are often ample—in other cases, large doses are absolutely essential—and in *extreme* cases the remedy may be pushed to full iodism by increasing a moderate dose slowly, till the point is reached. To this end it may be necessary to administer two drachms, and even half an ounce of the salt in twenty-four hours; it is only necessary to dilute the solution abundantly, and to note the effect each day.

It may be presumed that the system of each individual is capable of eliminating a certain given quantity of the salt in twenty-four hours—this quantity differing in different individuals. It is my belief that these cases of syphilis require a dose which will somewhat exceed the quantity a patient will ordinarily eliminate, in order to effect the hyperplasias, the lardaceous and gummy deposits, and perhaps even the hæmatopathia.

If it were not foreign to the subject-matter of these pages, I should proceed to show that, contrary to the advice of many syphilographers, this treatment can be applied to cases of primary and secondary syphilis, with singular success.

In the Obstetric Journal of last November (p. 570), Dr. Steiner, of Prague, compares the iodine and mercurial treatment for syphilis, and gives the preference to the latter, because, among other reasons, of the effect of the former "on general nutrition, and its occasional production of iodism." I have yet to see or hear of the first occasion when these incidents were not speedily superseded by discontinuing the remedy, and by the administration of tonics. In this particular, how unfavorable a contrast is suggested by the occurrence of ptyalism under a mercurial course, which often is *not* relieved by a suspension of the remedy.

Recommendations of Cundurango, lately used exclusively for cancer, have recently appeared over the signature of some South American physicians, who state that its chief virtues are displayed in the treatment of syphilis. I believe no experience of such a medication in this country has yet been published.

